# Granuloma-Related Hypercalcemia Secondary to Silicone-Induced Granulomatous Lymphadenopathy: A Case Report

**DOI:** 10.7759/cureus.106585

**Published:** 2026-04-07

**Authors:** Alhussein Alfaour, Swapnil Ganeshpure, Mohammed Nazim Kunduvalappil Thachankandy, Fatima Batool

**Affiliations:** 1 Geriatrics, Queen Mary's University Hospital, Harlow, GBR; 2 Internal Medicine, The Princess Alexandra Hospital NHS Trust, Harlow, GBR; 3 Geriatrics, Royal Berkshire Hospital, Reading, GBR; 4 Geriatrics, The Princess Alexandra Hospital NHS Trust, Harlow, GBR

**Keywords:** calcitriol-mediated hypercalcemia, foreign body granuloma, granulomatous hypercalcemia, hypercalcemia workup, silicone granuloma, silicone lymphadenopathy, silicone migration, silicone-related complications, vitamin d-mediated hypercalcemia, vitamin d metabolism

## Abstract

Granuloma-mediated hypercalcemia is an uncommon cause of parathyroid hormone-independent hypercalcemia and is usually associated with sarcoidosis, chronic infection, or other inflammatory disorders. Foreign-body granulomatous reactions caused by silicone are rare and may occur years after exposure. We report the case of a 77-year-old woman with recurrent hypercalcemia, generalized weakness, gait instability, nausea, abdominal discomfort, and weight loss. Biochemical evaluation showed suppressed parathyroid hormone, normal 25-hydroxyvitamin D, elevated angiotensin-converting enzyme, and inappropriately normal 1,25-dihydroxyvitamin D. Cross-sectional and functional imaging demonstrated paraoesophageal and right axillary lymphadenopathy. Mammography and targeted axillary ultrasound supported silicone migration, with the ultrasound showing a characteristic snowstorm appearance. Core biopsy confirmed breast tissue with fat necrosis and foreign-body giant cell reaction to silicone, with no evidence of malignancy. The overall findings supported silicone-induced granulomatous lymphadenopathy as the cause of parathyroid hormone-independent hypercalcemia. This case highlights the importance of taking a detailed prior implant or cosmetic exposure history when evaluating unexplained hypercalcemia.

## Introduction

Hypercalcemia is a common biochemical abnormality with a broad differential diagnosis. Measurement of parathyroid hormone (PTH) is the key initial step in distinguishing PTH-mediated from PTH-independent hypercalcemia [1]. When PTH is suppressed, the differential diagnosis includes malignancy, vitamin D-mediated disorders, granulomatous disease, endocrinopathies, medications, and other less common inflammatory causes [1].

Granuloma-mediated hypercalcemia occurs through dysregulated extrarenal conversion of 25-hydroxyvitamin D to 1,25-dihydroxyvitamin D by activated macrophages, leading to increased intestinal calcium absorption and suppression of endogenous PTH [1,2]. Although sarcoidosis is the classic cause, silicone-related granulomatous disease has also been described after cosmetic injections and breast implants [3-8]. We present a case of recurrent PTH-independent hypercalcemia caused by silicone-induced granulomatous lymphadenopathy, supported by characteristic imaging and biopsy confirmation.

## Case presentation

A 77-year-old White British woman presented with progressive generalized weakness, a sensation of feeling “not right,” gait instability with her legs “giving way,” mild nausea, intermittent abdominal pain, recurrent hypercalcemia, and weight loss. Her medical history included hiatus hernia, spondylolisthesis, hypertension, heart failure, depression, prior bilateral salpingo-oophorectomy, previous ankle fracture treated with open reduction and internal fixation, and previous silicone breast implants that had subsequently been removed. There was no documented history of nephrolithiasis, vitamin D supplementation, calcium supplementation, or other medications or supplements known to contribute to hypercalcemia. She was a retired shop assistant, a non-smoker, lived with her husband, and mobilized with a roller frame.

On examination, she appeared mildly frail but clinically stable with a National Early Warning Score 2 (NEWS2) score of 0 [9]. She was well perfused and hydrated. Cardiovascular and respiratory examination was unremarkable. Mild pedal edema and generalized abdominal discomfort were present, and no discrete palpable breast or axillary mass was identified.

All laboratory investigations are summarized in Table 1.

**Table 1:** Summary of laboratory investigations.

Parameter	Patient value	Reference range
Calcium	3.12 mmol/L	2.2-2.6 mmol/L
Adjusted calcium	3.32 mmol/L	2.2-2.6 mmol/L
Urea	17.9 mmol/L	2.5-7.8 mmol/L
Creatinine	173 μmol/L	53-97 μmol/L
eGFR	25 mL/min	60-150 mL/min
Albumin	30 g/L	35-50 g/L
Phosphate	1.37 mmol/L	0.8-1.5 mmol/L
Magnesium	0.6 mmol/L	0.7-1.0 mmol/L
PTH	0.6 pmol/L	1.2-12.0 pmol/L
25-hydroxyvitamin D	99 nmol/L	50-150 nmol/L
1,25-dihydroxyvitamin D	86 pmol/L	55-139 pmol/L
ACE	100 IU/L	16-85 IU/L
TSH	3.25 mU/L	0.35-5 mU/L
Serum electrophoresis/paraprotein	No paraprotein	-
AFP	2 U/mL	0-12 U/mL
CEA	< 2 μg/L	0-5 μg/L
CA-125	18 U/mL	0-35 U/mL
CA 15-3	25 U/mL	0-28 U/mL
Spot urine calcium	3.4 mmol/L	-
Spot urine creatinine	8.2 mmol/L	-
Spot urine calcium/creatinine ratio	0.41 mmol/mmol	0.2-0.6 mmol/mmol

Biochemical evaluation demonstrated PTH-independent hypercalcemia with renal impairment and hypoalbuminemia. The elevated serum angiotensin-converting enzyme (ACE) and the inappropriately normal 1,25-dihydroxyvitamin D level supported a granulomatous mechanism for hypercalcemia in the appropriate clinical context. Random urine calcium and creatinine reference ranges are laboratory-dependent; for interpretive context, the spot urine calcium/creatinine ratio was within the adult reference range.

**Figure 1:**
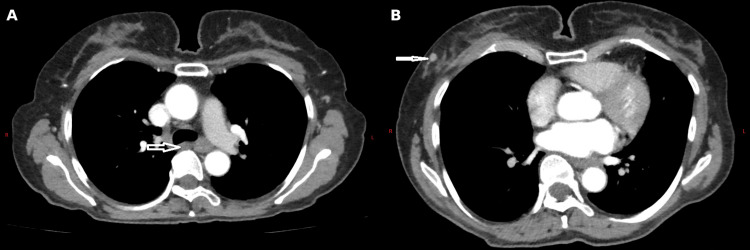
Contrast-enhanced computed tomography images showing (A) a paraoesophageal lymph node and (B) right axillary lymphadenopathy.

Computed tomography of the chest, abdomen, and pelvis showed small paraoesophageal lymph nodes and multiple right axillary lymph nodes, as illustrated in Figure 1.

**Figure 2:**
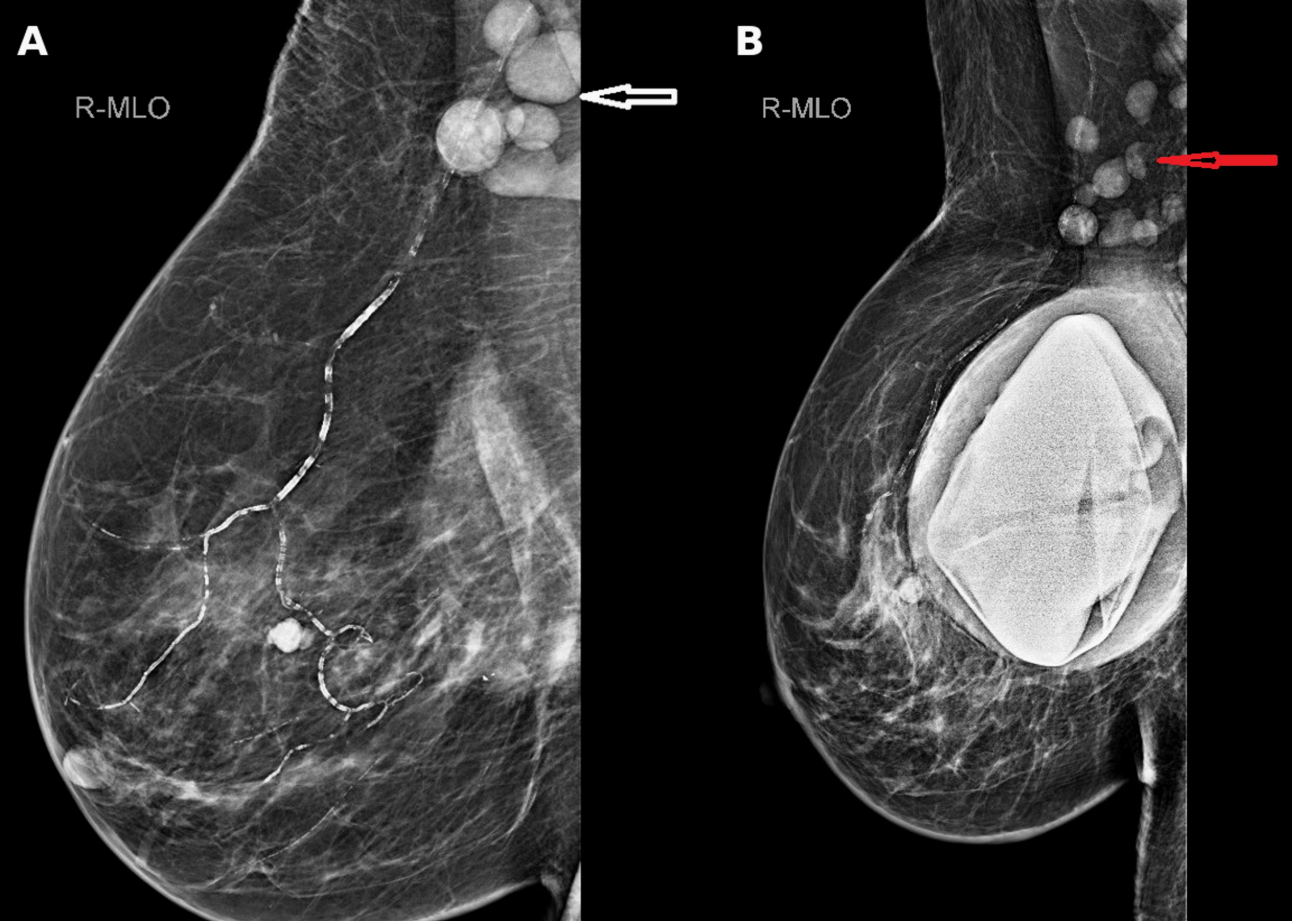
Mammographic appearances showing (A) right axillary nodes adjacent to the breast implant and (B) persistent opaque right axillary nodes on historical mammography.

Mammography and review of prior imaging showed dense right axillary nodes and changes related to previous silicone breast implants without a suspicious breast mass, as shown in Figure 2.

**Figure 3:**
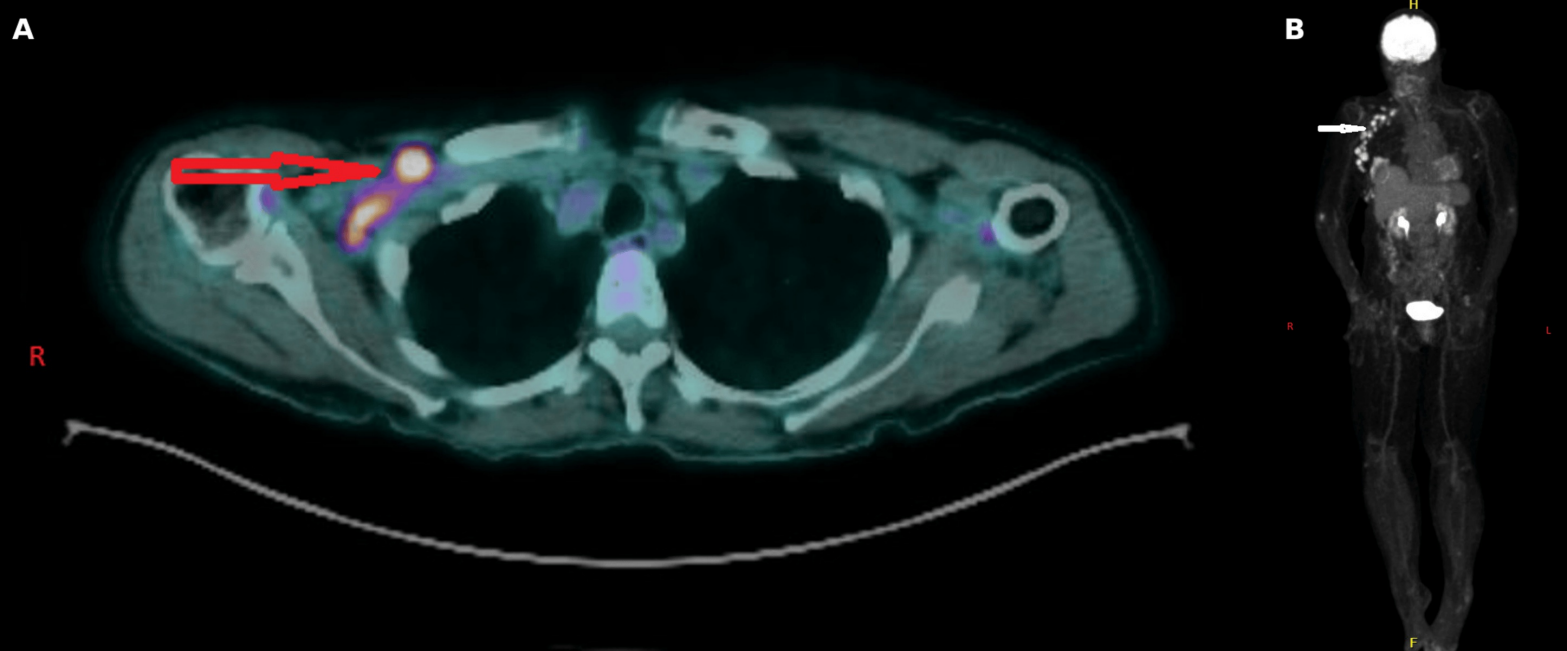
PET-CT images showing fluorodeoxyglucose (FDG)-avid right axillary lymph nodes on (A) fused axial imaging and (B) whole-body maximum intensity projection imaging.

Functional imaging with positron emission tomography-computed tomography demonstrated fluorodeoxyglucose (FDG)-avid right axillary lymphadenopathy, as shown in Figure 3.

**Figure 4:**
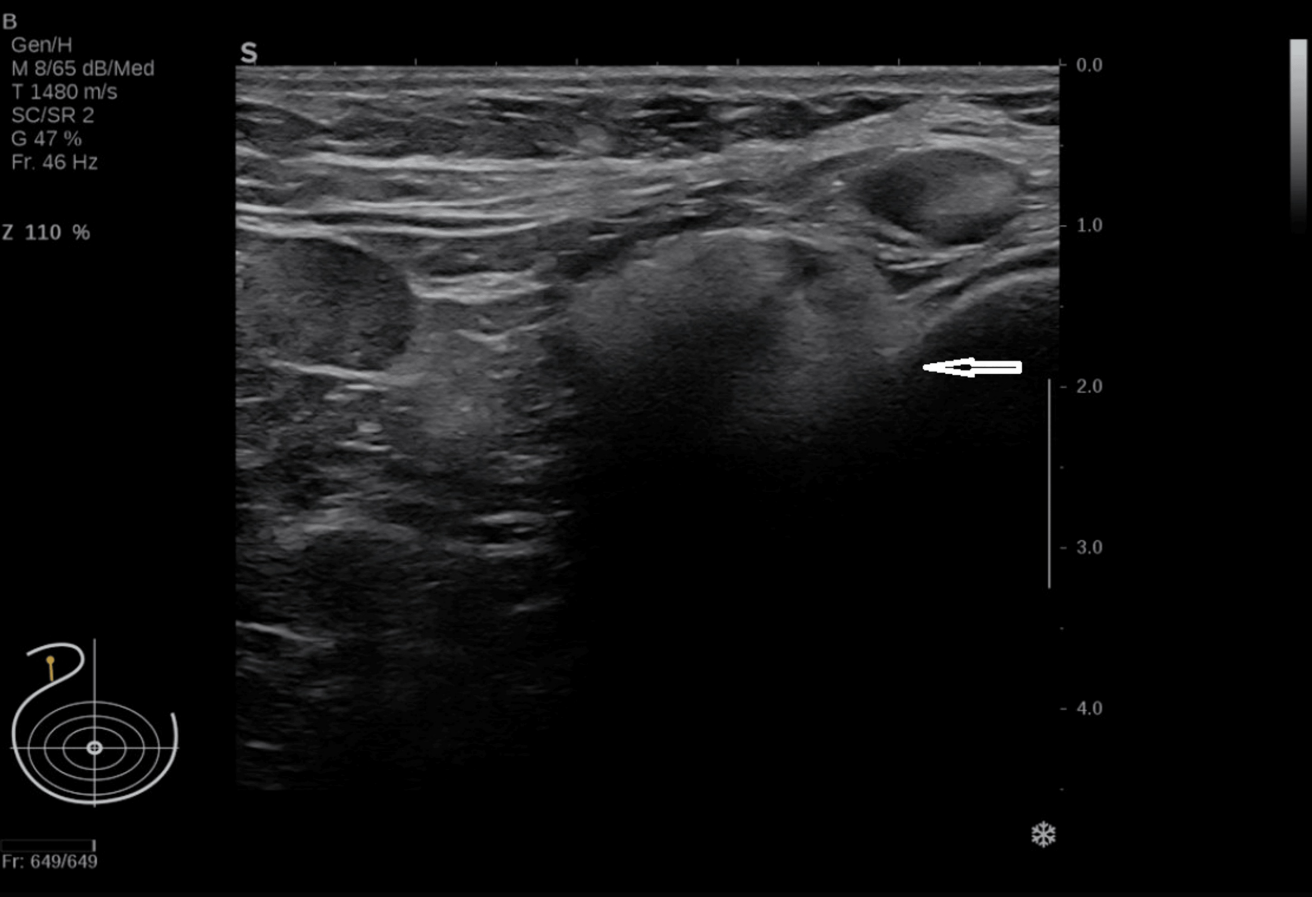
Targeted ultrasound of the right axilla demonstrating a characteristic “snowstorm” appearance, a sonographic finding consistent with silicone lymphadenopathy.

Targeted ultrasound of the right axilla demonstrated a characteristic “snowstorm” appearance strongly associated with silicone lymphadenopathy, as shown in Figure 4 [10,11].

Ultrasound-guided core biopsy of the right axillary abnormality showed breast tissue with fat necrosis and foreign-body giant cell reaction to silicone. No invasive carcinoma was identified. Taken together, the suppressed PTH, the biochemical pattern suggestive of granulomatous vitamin D activation, the imaging findings, the characteristic ultrasound appearance, and the biopsy result supported a diagnosis of silicone-induced granulomatous lymphadenopathy causing recurrent hypercalcemia [3-8,10,11].

The patient was treated with intravenous fluids, a bisphosphonate, and a short course of corticosteroid therapy. Her calcium improved to 2.53 mmol/L by discharge, and her symptoms resolved. Multidisciplinary discussion with endocrinology and breast surgery supported conservative management with outpatient monitoring. She remained under follow-up with both her general practitioner and the endocrinology clinic. At one-month follow-up, her calcium had normalized to 2.20 mmol/L. She had one further hospital admission two months after discharge with recurrent hypercalcemia, for which she was treated with another short course of corticosteroids and zoledronic acid. She required no further hospital admissions thereafter. Her latest follow-up on 02/03/2026 showed a calcium level of 2.49 mmol/L.

## Discussion

This case illustrates an uncommon but clinically important cause of PTH-independent hypercalcemia. Once PTH is suppressed, clinicians must rapidly evaluate for malignancy, vitamin D-mediated disease, and granulomatous disorders [1]. Silicone-associated granulomatous inflammation is rare, but the published literature shows that it can produce severe hypercalcemia and acute kidney injury, often months to years after prior silicone exposure [3-8].

The underlying mechanism is thought to parallel that seen in sarcoidosis. Activated macrophages within granulomatous tissue express extrarenal 1α-hydroxylase, increasing conversion of 25-hydroxyvitamin D to 1,25-dihydroxyvitamin D and thereby increasing intestinal calcium absorption [1,2]. In this setting, a 1,25-dihydroxyvitamin D level may remain within the laboratory reference interval yet still be inappropriate for the degree of hypercalcemia and PTH suppression. Elevated ACE may support granulomatous activity, but it is nonspecific and cannot establish the diagnosis in isolation.

The imaging in this case was particularly helpful. Silicone migration from breast implants can lead to deposition in regional lymph nodes and can mimic malignancy on computed tomography, mammography, and PET-CT [8,10,11]. The axillary ultrasound “snowstorm” sign has been shown to be highly specific for silicone lymphadenopathy and was a key clue in this patient [11]. Histology then excluded invasive carcinoma and confirmed a foreign-body giant cell reaction to silicone, allowing a unifying diagnosis.

Management of silicone-related granulomatous hypercalcemia is not standardized. Acute treatment includes hydration and antiresorptive therapy when indicated, while glucocorticoids are often used to suppress macrophage-mediated extrarenal calcitriol production [1,12]. Implant removal or further surgical excision may be considered in selected patients with implant rupture or a clearly identifiable, technically accessible silicone source; however, management should be individualized, as silicone migration and diffuse granulomatous disease may limit the benefit of surgery and make complete excision unfeasible [5,13]. In our patient, the implants had already been removed prior to presentation, and there was no clearly resectable residual target likely to eliminate the inflammatory burden completely. Following multidisciplinary discussion, conservative management with close outpatient monitoring was considered the most appropriate approach. In this patient, calcium normalized at one-month follow-up and remained controlled with ongoing endocrinology and primary care monitoring, although she required one brief readmission for recurrent hypercalcemia two months after discharge that responded to a further short course of corticosteroids and zoledronic acid. No subsequent hospital admissions were required.

## Conclusions

Silicone-induced granulomatous lymphadenopathy is a rare but important cause of PTH-independent hypercalcemia. A careful exposure history, structured biochemical assessment, targeted imaging, and histopathological confirmation are essential to distinguish this entity from malignancy and other granulomatous disorders. The presence of a snowstorm appearance on axillary ultrasound can provide an important diagnostic clue when silicone migration to lymph nodes is suspected.
